# In Vitro TLR4 Stimulating Bioactivities of Amylase/Trypsin-Inhibitors from Wheat (*Triticum aestivum* L.) Bred from 1891 to 2010

**DOI:** 10.3390/foods15091541

**Published:** 2026-04-29

**Authors:** Manjusha Neerukonda, Sabrina Geisslitz, Darina Pronin, Valentina Curella, Sibylle Neufang, Sandra Koch, Klajdi Begaj, Ernesto Bockamp, Heiko Weichert, Andreas Börner, Hans Weber, Katharina Anne Scherf, Detlef Schuppan

**Affiliations:** 1 Institute for Translational Immunology and Research Center for Immune Therapy, University Medical Center Mainz, 55131 Mainz, Germany; manjusha@uni-mainz.de (M.N.); valentinacur@hotmail.it (V.C.); sibylle.neufang@unimedizin-mainz.de (S.N.); sandra.koch@unimedizin-mainz.de (S.K.); klajdi.begaj@unimedizin-mainz.de (K.B.); bockamp@uni-mainz.de (E.B.); 2 Leibniz-Institute for Food Systems Biology at the Technical University of Munich, 85354 Freising, Germany; s.geisslitz.leibniz-lsb@tum.de (S.G.); darina.pronin@gmail.com (D.P.);; 3 Genebank Department, Leibniz Institute of Plant Genetics and Crop Plant Research, 06466 Seeland, Germany; weicherh@ipk-gatersleben.de (H.W.); weber@ipk-gatersleben.de (H.W.); 4 Department of Molecular Genetics, Leibniz Institute of Plant Genetics and Crop Plant Research, 06466 Seeland, Germany; boerner@ipk-gatersleben.de; 5 Food Biopolymer Systems, TUM School of Life Sciences, Technical University of Munich, 85354 Freising, Germany; 6 Division of Gastroenterology, Beth Israel Deaconess Medical Center, Harvard Medical School, Boston, MA 02215, USA

**Keywords:** cultivation, cultivar, genotype, environment, inflammation, gluten

## Abstract

Wheat amylase trypsin inhibitors (ATIs) are prominent allergens in Baker’s asthma and contribute to innate inflammation in non-celiac wheat sensitivity (NCWS), linking them to metabolic and autoimmune diseases. Their tetra-, di-, and monomeric forms, stabilized by disulfide bonds, confer resistance to digestion, baking, and heating. Although proteomic studies reveal minor variation in ATI subtypes among cultivars and major variation among species, the influence of environment and wheat genotype on ATI levels and TLR4-stimulating activity remains unclear. We assessed the effect of the environment on the in vitro inflammatory bioactivity of ATIs extracted from 60 German wheat genotypes focusing on breeding over time between 1891 and 2010, and cultivation across three climatically distinct years. We found considerable genotype-dependent variation in ATI bioactivity that did not correlate with ATI subtype abundance, and observed no consistent difference between old and modern cultivars. ATIs from samples grown in 2019, a warm and dry year, showed reduced TLR4 activity, highlighting the significant impact of environmental conditions on inflammatory ATI bioactivity.

## 1. Introduction

Wheat is a staple food providing essential nutrients but wheat can also trigger immune-mediated disorders such as celiac disease, classical wheat allergy, and non-celiac wheat sensitivity (NCWS), characterized by innate and type 2 allergic immune responses, and affecting up to 10% of wheat consuming populations [1,2,3,4,5]. Amylase/trypsin-inhibitors (ATIs), a family of 17 non-gluten proteins, are important triggers of NCWS through the activation of toll-like receptor 4 (TLR4) expressed on intestinal immune cells [6,7,8]. ATIs exist as mono-, di-, and tetrameric isoforms, with tetrameric CM types (e.g., CM3, CM16) and dimeric 0.19/0.28 forms showing the highest TLR4 stimulating bioactivity. Biochemical, mass spectrometry, and cell-based studies confirm ATIs but not gluten as the primary drivers of wheat’s innate immune effects [6,7,9].

Notably, dietary ATIs are resistant to heat treatment and intestinal digestion, and promote intestinal and systemic inflammation, exacerbating multiple chronic intestinal and extra-intestinal diseases including inflammatory bowel disease (IBD), celiac disease, metabolic syndrome/steatohepatitis, classical allergies, multiple sclerosis, and neuroinflammation in mouse models [1,6,7,8,10,11,12,13,14], and these findings were confirmed in controlled clinical studies of patients on a wheat-free vs. wheat-containing diet [15,16,17,18].

Our prior proteomic studies using liquid chromatography tandem mass spectrometry (LC-MS/MS) showed significant differences in total ATI content and ATI subtypes across old towards modern wheat species, across three locations. Einkorn had low ATI content, and mainly dimeric 0.19 subtype and CMX1/2/3 compared to common hexaploid and tetraploid wheat. Emmer and durum were dominated by ATIs CM3, CM2, CM16, and 0.53 [19,20]. QconCAT-based MS quantification across 60 cultivars reinforced these interspecies differences, with einkorn lacking several ATI isoforms such as CM17 [21]. While 0.19 and CM3 are established TLR4 ligands [6,22], the detectable TLR4-activating species of einkorn remain unclear [19]. Overall, wheat digestibility varies with composition but shows no clear distinction between old and modern varieties registered before and after 1960 [23].

Environmental and genetic factors also influence ATI levels. Studies of durum wheat across multiple years and locations revealed significant variation in CM3 ATI, driven by genotype and environment [24,25]. Major ATI diversity was documented in common wheat cultivars via epitope mapping and LC-MRM-MS [26], with 7–34% variation found among German and Turkish hexaploid wheat varieties [27]. Genetic analyses of European wheat cultivars showed 2.5- to 6-fold differences in ATI subtype levels linked to cultivar and environment [28]. ATIs, especially CM1, CM3, and CM17, dynamically respond to drought and heat stress through changes in abundance and post-translational modifications, highlighting their role in wheat stress adaptation [29].

In this study, we investigated how genetic variation (old vs. modern cultivars within common wheat (*Triticum aestivum*)) and environmental factors (three crop years: 2015, 2017, 2019) affect ATI-induced TLR4 bioactivity using a HeLa dual TLR4 reporter cell line, aiming to clarify the combined impact of cultivar and growing conditions on wheat ATI immunogenicity. In our previously reported study using the same sample set, we quantified 13 ATIs in 60 wheat cultivars (1891–2010) via targeted LC-MS/MS. Total ATIs, major ATIs (0.19, CM3), and overall ATI distribution remained unchanged [30]. In the current study, we determined TLR4 stimulating ATI bioactivities, a key contributor to inflammation in NCWS, in the 60 cultivars grown in 2015, 2017 and 2019, and correlated them with ATI and ATI subtype quantities as determined by LC-MS/MS, to find a clear correlation with environmental conditions vs. mere ATI quantity.

## 2. Methods

### 2.1. Grain Samples

The data on cultivation conditions and genotypes were reported in our prior study [30]. Briefly, sixty hexaploid wheat (*Triticum aestivum* L.) cultivars were selected from the German Federal ex situ Genebank at the Leibniz Institute of Plant Genetics and Crop Plant Research (IPK, Gatersleben, Germany) (Supplementary Table S1). These cultivars represent the five most widely grown wheat varieties in Germany, registered per decade between 1891 and 2010. Based on registration dates, cultivars registered prior to 1950 (samples 1–30) were classified as “old,” while those registered from 1950 onward (samples 31–60) were designated as “modern.” The cultivars were grown under field conditions in Gatersleben during three crop years: 2015, 2017, and 2019. For each genotype and year, grains from three biological replicate plots were harvested and pooled. During these years, the mean temperatures recorded were 10.2 °C, 10.0 °C, and 10.8 °C, while total precipitation measured 533 mm, 557 mm, and 386 mm in 2015, 2017, and 2019, respectively (https://wetter.ipk-gatersleben.de/, accessed on 20 January 2026).

The classification of cultivars into “old” and “modern” reflects key phases in the development of wheat breeding in Germany and provides essential historical context for interpreting the results. Early cultivars of Triticum aestivum, registered before 1950, were primarily developed through phenotypic selection from landraces and locally adapted material, with limited use of controlled crosses and minimal emphasis on yield optimization or uniformity. In contrast, wheat breeding in Germany after the mid-20th century became increasingly systematic and science-based, incorporating controlled hybridization, genetic selection, and targeted breeding objectives such as higher yield, reduced plant height, improved disease resistance, and suitability for mechanized and higher-input agricultural systems [31]. These developments were part of broader agricultural advances associated with the Green Revolution. Consequently, “modern” cultivars represent germplasm shaped by intensive breeding programs, whereas “old” cultivars reflect earlier breeding approaches with greater genetic heterogeneity and adaptation to lower-input conditions.

### 2.2. ATI Extraction

For ATI extraction, 1 g of flour ground from pooled grains per genotype and year was extracted twice consecutively with 5 mL of 10 mM Tris, 0.5 M NaCl, pH 7.8, following a modified published method (7,9). The supernatants from both extractions were combined. To exclude lipopolysaccharide (LPS) contamination, samples were pre-adsorbed with Pierce™ High-Capacity Endotoxin Removal Resin (Thermo Fisher Scientific, Rockford, IL, USA) at a 2:1 sample-to-resin ratio by constant spinning for 1 h at 4 °C, followed by centrifugation at 500× *g* for 1 min to collect the clarified extracts.

### 2.3. TLR4 Stimulating Bioactivity Determination of ATIs

Stably transfected HeLa TLR4/MD-2/CD14/IL-8 Prom/LUCPorter™ Renilla luciferase reporter cells (Novus Biologicals, Wiesbaden, Hessen, Germany) were further co-transfected with a CMV promoter-driven firefly luciferase reporter gene under the control of the IL-8 promoter, enabling the simultaneous quantification of TLR4 activation and cell viability, as described (7,9). HeLa-TLR4 dual reporter cells (20,000 cells/well) were seeded in 100 μL DMEM supplemented with hygromycin (InvivoGen, Toulouse, France) (125 μg/mL) in 96-well flat-bottom plates and allowed to adhere for 24 h. Cells were stimulated in duplicate with 0.625–5 μL ATI extract in 100 μL DMEM (Sigma Aldrich, Saint Louis, MO, USA) for 7 h. LPS (Sigma Aldrich, Saint Louis, MO, USA) (0.5–2 ng/100 μL) served as a positive control, plain medium as a negative control, and modern hexaploid wheat extract as an internal standard. After stimulation, cells were washed with PBS and lysed with 10 μL/well passive lysis buffer (Promega, Walldorf, Baden-Württemberg, Germany) by freeze–thawing. Subsequently, 50 μL LAR II solution (Promega) was added, and 40 μL of the lysate was transferred to a white plate to measure Firefly luminescence, followed by the addition of 40 μL Stop and Glo buffer to measure Renilla luminescence. Both signals were recorded using a Tecan Reader (Männedorf, Switzerland). Normalized TLR4 activity was calculated by dividing the Renilla (TLR4-driven) signal by the firefly (constitutive) signal and further normalized to LPS-induced TLR4 activity. ATI bioactivities were determined based on the wheat extract standard curve equivalent to 3 mg of bioactive ATI protein per g of dried standard wheat flour.

### 2.4. Data Analysis

Two-way analysis of variance (ANOVA) with the factors genotype and environment (harvest year) and interaction enabled was performed in Origin 2023 (OriginLab Corporation, Northampton, MA, USA). Prior to ANOVA, the normality of the data distribution was assessed using the Shapiro–Wilk test and the homogeneity of variances was examined using Levene’s test. Pearson correlations at a significance level of *p* < 0.05 were also done in Origin 2023. Correlation analysis between ATI TLR4 stimulating bioactivity and ATI quantities was performed using GraphPad Prism 9 software. The correlation analysis was performed using a predefined significance level of α = 0.05. Correlation analyses were conducted using the Pearson product–moment correlation coefficient (Pearson’s r). For each correlation, the correlation coefficient (r), 95% confidence interval (CI), coefficient of determination (R^2^), and corresponding *p* values (two-tailed) were calculated. Statistical significance was determined based on α = 0.05.

## 3. Results

### 3.1. Samples and Bioactivity Assessment

We evaluated the innate immune activation potential of quantitative flour extracts from 30 old and 30 modern cultivars of *Triticum aestivum* (registered before and after 1950, respectively) cultivated during three climatically different harvesting years (for sample information see Supplementary Table S1) using a HeLa dual TLR4 reporter cell line. Correlation analysis was performed between the quantity of ATI subtypes of each wheat genotype and the environmental conditions versus the TLR4 stimulating ATI bioactivities.

### 3.2. Determination of TLR4 Stimulating Bioactivities of 60 Wheat Genotypes Grown Under Three Different Climatic Conditions in 2015, 2017, and 2019

**Figure 1:**
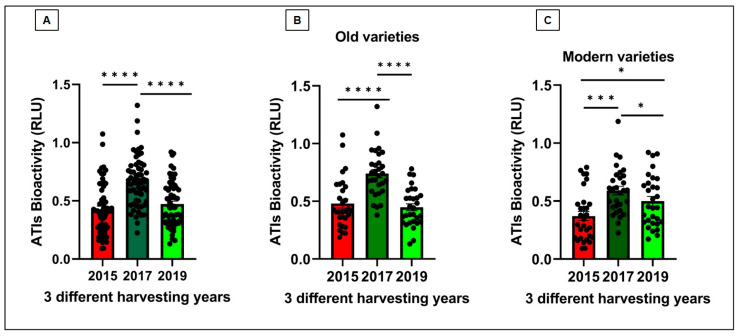
(**A**) TLR4 stimulating bioactivity of 60 old and modern wheat genotypes in total per each crop year 2015, 2017, and 2019, tested on our Hela TLR4 dual reporter cell line. Each column represents mean of 60 genotypes. (**B**,**C**) TLR4 stimulating bioactivity of 30 old and 30 modern wheat genotypes per each crop year 2015, 2017 and 2019 tested on our Hela TLR4 dual reporter cell line and compared. Each column represents mean of 30 genotypes. RLU—Relative Luminescence Units. Significance levels—* *p* < 0.05; *** *p* < 0.001, **** *p* < 0.0001.

**Figure 2:**
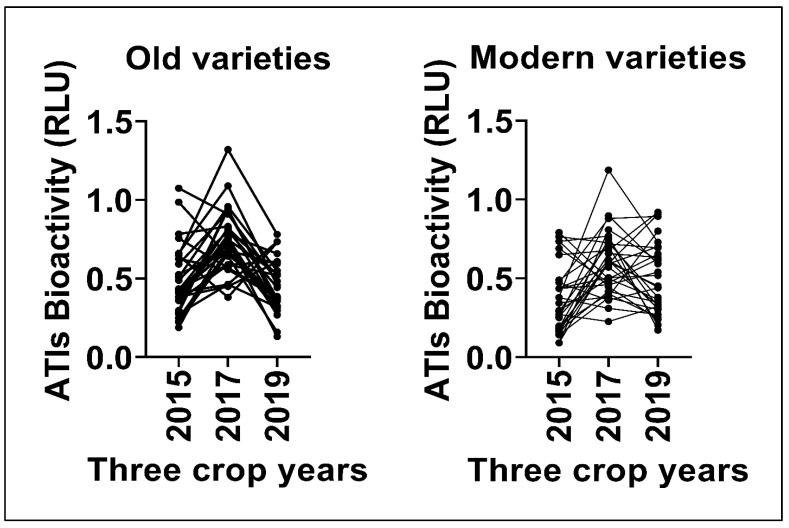
Plots representing TLR4 stimulating bioactivity of 30 old and 30 modern wheat genotypes per each crop year 2015, 2017, and 2019 tested on our Hela TLR4 dual reporter cell line and compared. RLU—Relative Luminescence Units.

**Figure 3:**
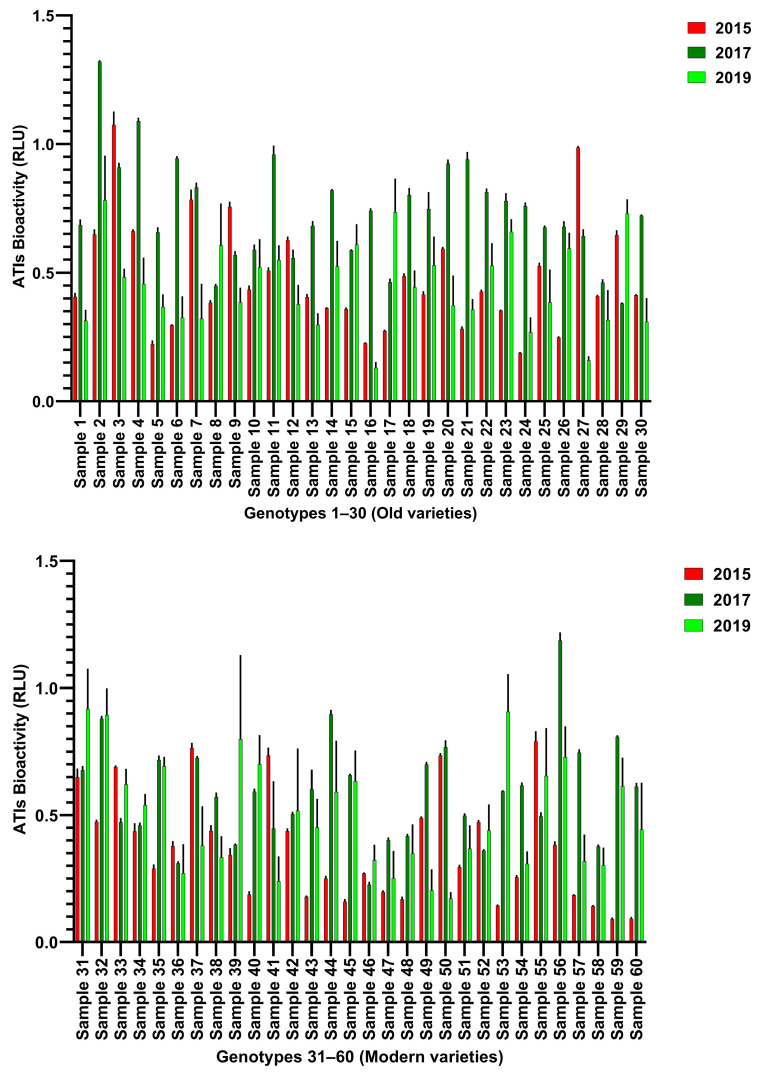
TLR4 stimulating bioactivity of all individual 60 old and modern wheat genotypes per each crop year 2015, 2017, and 2019 tested on our Hela TLR4 dual reporter cell line and compared. RLU—Relative Luminescence Units. Gray lines represent standard error of mean (SEM).

The hexaploid wheat cultivars were grown under three different climatic conditions in the years 2015, 2017, and 2019 in Gatersleben (Germany). Figure 1A shows an overall increase in TLR4 stimulating bioactivities of all 60 samples in the year 2017 followed by 2015 and 2019. All bioactivity data corresponding to each harvest year and wheat genotype are specified in Supplementary Table S2 and Figure 2. Figure 1B illustrates the mean bioactivities of samples 1–30 (old varieties) grown over three years, following a pattern similar to that presented in Figure 1A. In contrast, Figure 1C illustrates the mean bioactivities of the modern varieties (31–60) that displayed a slight increase in mean bioactivity in 2019 compared to 2015, the opposite of the trend observed in old varieties. Nevertheless, bioactivity peaked in 2017 for both old and modern genotypes. Comparison of old and modern wheat genotypes (Figure 1B,C) shows that old varieties are not considered less safe than modern varieties. Figure 2 and Figure 3 show the comparison of all 60 individual genotypes grown in 2015, 2017 and 2019 which correspond to the above-mentioned data in Figure 1A–C. One modern variety, Dekan, showed low bioactivities in all 3 years (2015, 2017 and 2019: RLU: 0.14, 0.37 and 0.30, respectively).

### 3.3. Effect of Genotype and Environment on TLR4 Stimulating Bioactivity

**Figure 4:**
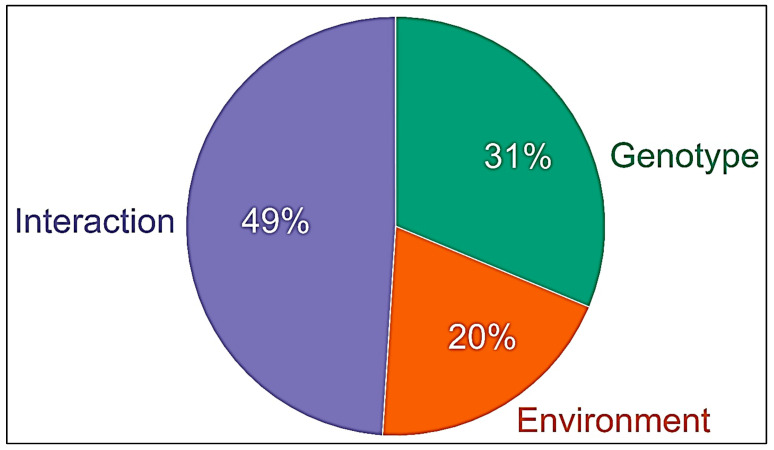
Influence of genotype, environment, and their interaction on the TLR4 stimulating bioactivity of ATIs. Data analysis was performed using 60 old and modern wheat genotypes per year 2015, 2017, and 2019.

The results of the two-way ANOVA show the effect of the genotype, environment, and their interaction on TLR4 stimulating bioactivity (Figure 4). The effect of the genotype (31%) was higher than that of the environment (20%), but the main effect was due to the interaction of genotype and environment (49%), showing that both factors influence one another to a large extent and cannot be clearly separated.

### 3.4. Correlation Analysis Between TLR4 Stimulating Bioactivity and Quantity of Total ATIs and ATI Subtypes

**Figure 5:**
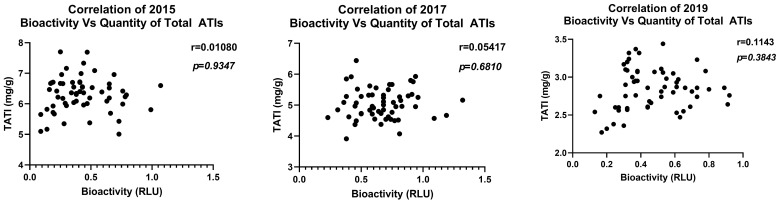
Correlation analysis between bioactivity of ATIs from three crop years versus quantity of total 13 ATI subtypes. TATI—Total ATIs. N = 60 per crop year.

**Figure 6:**
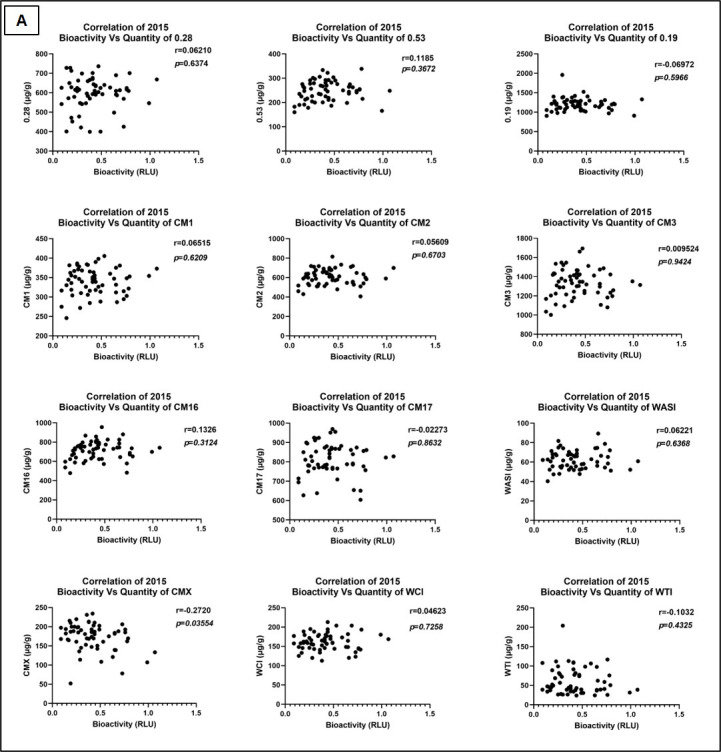

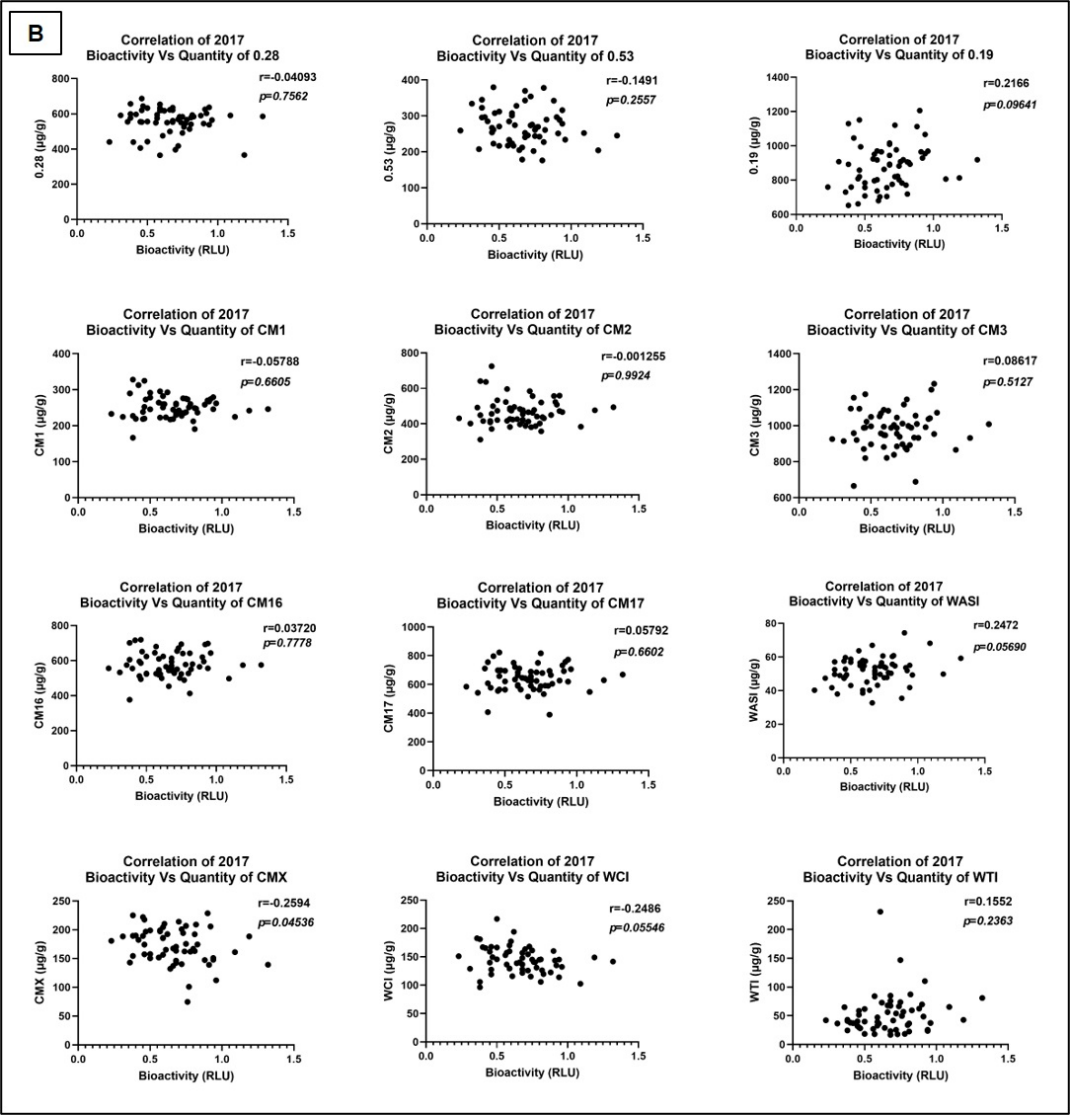

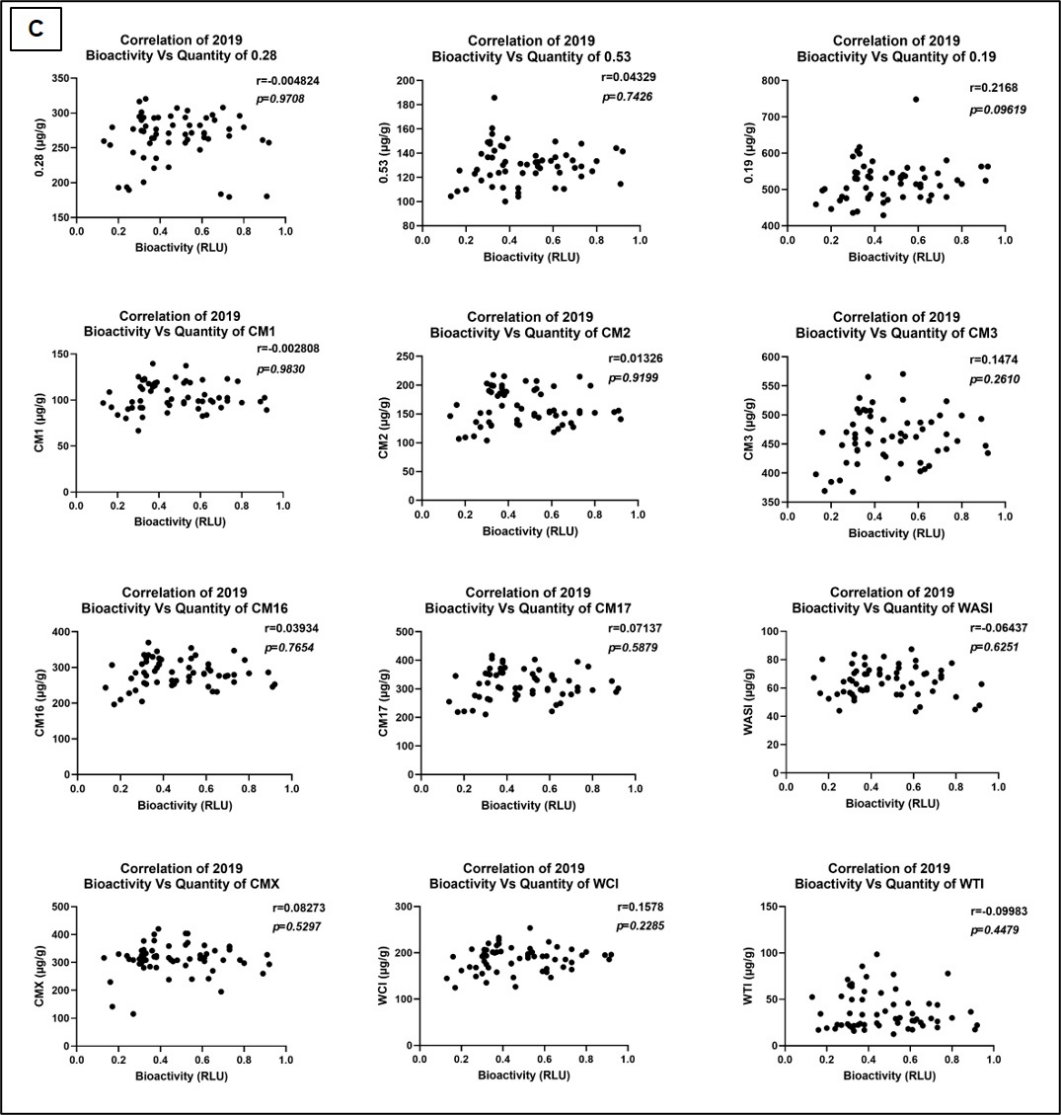
Correlation analysis of TLR4 stimulating bioactivity in crop year 2015 (**A**), 2017 (**B**) and 2019 (**C**) versus individual ATI subtypes. RLU—Relative Luminescence Units, WASI—wheat amylase subtilisin inhibitor, WTI—Bowman–Birk type trypsin inhibitor and WCI—wheat chymotrypsin inhibitor.

Comparison of ATI bioactivity in each of the three crop years versus quantity of total ATIs (mean of 13 ATI subtypes) showed no significant correlation (Figure 5). Correlation analysis was also performed between bioactivity and single ATI subtypes for each crop year (Figure 6). Here, a significant negative correlation of CMX ATI with bioactivity in the years 2015 and 2017 was found. All data corresponding to LC-MS/MS quantification of ATIs of each crop year is provided in Supplementary Tables S3–S5.

## 4. Discussion

In this study, we measured disease-relevant TLR4-stimulating bioactivities associated with wheat ATIs in flours of 30 old and 30 modern hexaploid wheat cultivars grown over three crop years (2015, 2017, and 2019). Moreover, we evaluated the influence of wheat genotype, environment, and their interaction on TLR4 stimulating bioactivity of ATIs. Our results reveal significant variation in ATI bioactivity (Figure 1, Figure 2 and Figure 3) driven by both genetic background and environmental conditions as well as interaction effects between both (Figure 4), highlighting for the first time how these factors interplay to modulate wheat’s innate immune-activating potential.

Previous studies have shown that wheat properties including yield and protein composition vary between older and modern hexaploid varieties, with environmental factors such as drought, temperature, and fertilization further affecting these traits under standard cultivation conditions. In addition, protein content and baking quality are sensitive to nitrogen fertilization regimes, which also influence the expression of specific ATI subtypes, as shown for *Triticum aestivum* [32,33]. These changes in ATI expression and functionality can affect the nutritional quality, functional properties of wheat, including dough extensibility and gluten formation.

Regarding ATI expression, our study confirms prior findings of genotype-specific variation in ATI subtypes and quantities. For instance, dimeric ATIs such as 0.19 and 0.28 and tetrameric CM-type ATIs differ markedly among wheat species and cultivars, with these differences further modulated by environmental conditions (19, 21). Notably, our current data suggest that environmental factors that prevail in different crop years also influence ATI bioactivity (Figure 4) beyond mere protein quantities (Figure 5). This is consistent with reports on environmentally induced protein nitration, a post-translational modification that can modulate protein functionality [33], and which also enhanced ATI bioactivity [34].

Importantly, our study highlights that ATI quantity alone does not reliably predict TLR4 bioactivity (Figure 5 and Figure 6), except for a minor but a significant negative correlation of CMX ATI subtype with bioactivity in the crop years 2015 and 2017 (Figure 6). Instead, genotype-specific differences in ATI composition, and possibly processing during growth, determine the immune-activating potential of the ATI-containing wheat extracts. This is underscored by the low bioactivity of the Dekan cultivar (Figure 3 and Supplementary Table S2), which consistently showed reduced ATI content [30], and here also reduced TLR4 activation across three years. Interestingly, environmental stressors, especially elevated temperatures and dry conditions recorded in 2019, appear to exert a major influence on ATI bioactivity decreasing TLR4 stimulating potential, likely through alterations in general protein expression but also modification, subsequent to limited water availability. That post-translational and flour processing related modifications like sourdough fermentation can significantly reduce ATI protein quantity as well as inflammatory bioactivity has previously been demonstrated [35,36].

These findings support the notion that ATI bioactivity results from a complex interplay between quantity, isoform composition, post-translational modifications, including nitration, or other environmental agents [34], and potentially interactions with other wheat components. This complexity is further supported by robust evidence from cell culture models and in vivo studies demonstrating the relevance of ATI bioactivity to innate immune activation and inflammatory disease exacerbation in both animal experimental models and patients [1,6,7,8,10,11,12,13,14].

## 5. Conclusions

This study demonstrates that both wheat genotype and environmental conditions, as well as their interaction, play a critical role in shaping the in vitro TLR4-stimulating bioactivity of wheat amylase trypsin inhibitors (ATIs). Climatic variation had a significant impact, with all cultivars showing markedly increased proinflammatory activity in 2017 compared to years 2015 and 2019, highlighting the importance of environmental factors in modulating ATI-related immune activation potential. Importantly, no consistent difference in bioactivity was observed between old and modern Triticum aestivum cultivars, indicating that older varieties cannot be considered inherently safer with respect to ATI-related proinflammatory effects. Instead, substantial variability was detected among individual genotypes, suggesting that genotype-specific traits, rather than breeding era, are more relevant determinants of ATI bioactivity. Furthermore, the lack of a straightforward relationship between ATI quantity, as determined by mass spectrometry, and TLR4 activation, suggests that additional factors such as ATI subtype composition as mono- vs. oligomers, post-translational modifications, proteolytic processing, and interactions with other wheat components determine inflammatory bioactivity.

Overall, these findings emphasize the need to move beyond simplistic explanations, such as comparison between old and modern wheats, using standard proteomic analysis or genotypes alone, and focus on identifying low inflammatory wheats using bioactivity selection, furthering the definition of beneficial environmental conditions and wheat processing methods. Such insights can provide a foundation for targeted breeding combined with optimized cultivation and processing strategies aimed at producing healthy wheat products for patients with non-celiac wheat sensitivity (NCWS).

## Data Availability

The original contributions presented in this study are included in the article/Supplementary Materials. Further inquiries can be directed to the corresponding author.

## Supplementary Materials

The following supporting information can be downloaded at: https://www.mdpi.com/article/10.3390/foods15091541/s1,Table S1: Sample information; Table S2: Bioactivity data; Table S3: 2015 Bioactivity–ATIs quantity; Table S4: 2017 Bioactivity–ATIs quantity; Table S5: 2019 Bioactivity–ATIs quantity.
